# Progressive privacy-preserving batch retrieval of lung CT image sequences based on edge-cloud collaborative computation

**DOI:** 10.1371/journal.pone.0274507

**Published:** 2022-09-15

**Authors:** Yi Zhuang, Nan Jiang

**Affiliations:** 1 College of Computer & Information Engineering, Zhejiang Gongshang University, Hangzhou, P.R. China; 2 Affiliated Hangzhou First People’s Hospital, Zhejiang University School of Medicine, Hangzhou, P.R. China; Al-Balqa Applied University Prince Abdullah bin Ghazi Faculty of Information Technology, JORDAN

## Abstract

**Background:**

A computer tomography image (*CI*) sequence can be regarded as a time-series data that is composed of a great deal of nearby and similar *CI*s. Since the computational and I/O costs of similarity measure, encryption, and decryption calculation during a similarity retrieval of the large *CI* sequences (*CIS*) are extremely high, deploying all retrieval tasks in the cloud, however, will lead to excessive computing load on the cloud, which will greatly and negatively affect the retrieval performance.

**Methodologies:**

To tackle the above challenges, the paper proposes a progressive privacy-preserving *B*atch *R*etrieval scheme for the lung *CIS*s based on edge-cloud collaborative computation called the *BRS* method. There are four supporting techniques to enable the *BRS* method, such as: 1) *batch similarity measure for CISs*, 2) *CIB-based privacy preserving scheme*, 3) *uniform edge-cloud index framework*, and 4) *edge buffering*.

**Results:**

The experimental results reveal that our method outperforms the state-of-the-art approaches in terms of efficiency and scalability, drastically reducing response time by lowering network communication costs while enhancing retrieval safety and accuracy.

## Introduction

**Fig 1:**
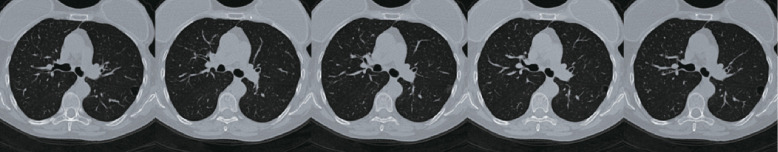
An example of a lung CIS with 5 neighboring *CI*s.

With the rapid growth of the number of medical images and the increasing demand for remote diagnosis, content-based mobile retrieval for *C*omputed tomography *I*mage *S*equences (*CIS*)s in telemedicine systems (*TS*s) [1] plays an increasingly important role in disease diagnosis in recent years. Fig 1 illustrates an example of a lung *CIS* consisting of a large number of nearby and visually similar *C*omputed tomography *I*mage(*CI*)s. As one of the main tasks of the *TS*, mobile-terminal-based high-resolution *CIS* retrieval enables medical professionals to identify lesion tissues in aberrant *CI*s and carry out the computer-assisted diagnosis and treatment.

The motivations of the *CIS* retrieval in edge-cloud collaborative computing mode are based on the following key observations:

- Instead of using the whole *CIS*, traditional *CI* retrieval takes a single *CI* as the retrieval one to perform similarity comparison which is ineffectual and inadequate in modeling the whole retrieval *CIS* leading to poor retrieval precision ratio;
- As the *CIS* data belongs to patients’ personal privacy information [2], it should be encrypted during the retrieval processing; otherwise, the personal information leakage will take place;
- To better understand the condition of their patients during remote consultations, doctors will frequently retrieve and examine their *CIS*s in real time, which involves high computational costs, as well as the intensive transmission of the *CIS*s. Therefore, deploying and executing all expensive retrieval and computing tasks in the cloud will result in significant computational overhead and have a negative impact on the retrieval’s performance improvement. To efficiently reduce the load of cloud computing, edge computing [3] came into being. As a new distributed computing mode, edge computing makes up for some shortcomings of cloud computing and diverts most computing tasks to edge device nodes (i.e., *edge server* (ES)) around the mobile terminal. This can not only significantly reduce the computing load on the cloud, but also reduce the transmission cost [4, 5] to support the retrieval in real time [6];
- For these mobile terminals whose computing resources are constraint such as the battery reserves, screen resolutions and computational powers, etc. The data transmission is negatively affected by the unstable network bandwidth which causes delays in the data retrieval and transmission, especially in rural areas with inadequate mobile communication infrastructure [1].

Based on the above analysis, the paper presents a privacy-preserving *B*atch *R*etrieval method for large lung *CIS*s in the edge-cloud computing network, called *the BRS*, by analyzing the similarity of the nearby *CI*s in the sequence. There are few studies on how to speedup the batch similarity retrieval of the large *CIS*s using the edge-cloud collaborative computing environment. Specifically, when a user submits a retrieval *CIS*(*X*_*R*_), firstly, an index mechanism at the edge layer called *eIndex* is used to quickly judge whether there are some answer *CIS*s similar to *X*_*R*_ in the edge buffer. If exists, then a high-dimensional similarity retrieval of the partial answer ones supported by the *cIndex* is carried out by accessing the cloud; otherwise, the similarity retrieval of all *CIS*s supported by the *cIndex* is performed directly through the cloud; finally the answer *CIS*s are returned to the user node. The extensive experiments demonstrate the effectiveness, efficiency, and scalability of the *BRS* method.

## Background

Over the past fifty years, content-based image retrieval(CBIR) has been a persistent and difficult research problem [6–10]. Due to the ‘*semantic gap*’, however, the retrieval accuracies are still not satisfactory.

As one of the key subfields of the CBIR, content-based medical image retrieval (CBMIR) research has become increasingly popular in recent years. The first CBMIR system built for high-resolution lung *CI*s is ASSERT [11]. After that, many prototype systems were developed, including IRMA [12], FIRE [13], and others. A noisy image bag-based technique for retrieving medical images was presented by Huang et al. [14]. To further reduce the ‘*semantic gap*’, Huang et al. [15] developed a relevance feedback technique for the CBMIR based on a noisy-smoothing model. Kitanovski et al. [16] designed a multi-modality-based CBMIR system. Lan et al. [17] proposed a simple texture feature extraction algorithm for the CBMIR. A multi-panel medical image segmentation framework for the CBMIR system was supplied by Ali et al. [18]. Based on the fusion of the wavelet optimization and adaptive block truncation coding, Kasban et al. [19] built a reliable CBMIR system. Tuyet et al. [20] used the deep learning techniques to support the salient region-based CBMIR.

Since the aforementioned CBMIR systems are based on single-PC mode, their retrieval performances are not satisfactory when dealing with a great deal of medical images [21]. Anbarasi et al. [22] developed a distributed CBMIR system using distributed database techniques. Charisi et al. [23] designed a parallel CBMIR scheme in a peer-to-peer(P2P) network. Based on the hybrid features, Depeursinge et al. [24] proposed a mobile access approach to peer-reviewed medical information. Although Zhuang et al. [25] put forward an efficient and robust CBMIR technique in a mobile wireless network, the retrieval efficiency is poor since the effectiveness of the load balance strategy needs to be further improved. Based on the previous work [25], Zhuang et al. [26] introduced a high-performance batch retrieval technique for medical images in wireless network from a standpoint of multi-retrieval optimization to further improve the retrieval efficiency. A mobile teleradiology system [27] is appropriate for streamlining the CBMIR procedure. For telemedicine applications, Chitra et al. [28] suggested an enhanced retrieval approach for brain images utilizing carrier frequency offset adjusted OFDM technique. To solve the ‘*semantic gap*’, Jiang et al. [29] introduced a novel framework of mobile similarity retrieval of medical images based on a crowdsourcing model.

On the basis of the *CI* analysis, Lei et al. [30] developed a sparse CNN model-based high-resolution *CI* retrieval technique. Yu et al. [31] presented a liver *CI* retrieval algorithm based on a non-tensor product wavelet. Based on an adder combining two local bit plane-based dissimilarities, Hatibaruah et al. [32] introduced a novel *CI* retrieval approach. Hwang et al. [33] applied a CBIR and CNN techniques to enable diffuse interstitial lung disease retrieval. To facilitate the effective diagnosis of the lung cancer, Alzubi et al [34] designed a boosted neural network ensemble classification approach.

Despite extensive study of the *CI* retrieval, the majority of approaches still rely solely on this retrieval without taking the *CIS* retrieval into account. Meanwhile, very little research has addressed *CIS* retrieval in the collaborative edge-cloud environment.

## Preliminaries and preprocessing

Firstly, the main symbol notations are listed in Table 1.

**Table 1:** Main notations used throughout the paper.

Notation	Meaning
*N*_*U*_	user node
*N*_*E*_	edge node
*N*_*C*_	cloud node
Ω	a set of *n* *CIS*s in *N*_*C*_
Ω′	the *CIS*s buffered at *N*_*E*_
*X*_*i*_	the *i*-th *CIS* and *X*_*i*_ ∈ Ω
*X*_*R*_	the retrieval *CIS*
*r*_*R*_	the retrieval radius
Ψ	the final answer *CIS*s
Ψ′	partial answer *CIS*s based on the *eIndex* at *N*_*E*_
Ψ″	partial answer *CIS*s based on the *cIndex* at *N*_*C*_
$X_{R}^{\prime i}$	the *i*-th historical retrieval *CIS*
$r_{R}^{\prime i}$	the *i*-th historical retrieval radius
*CI*_*j*_	the *j*-th *CI* in *X*_*i*_ and *CI*_*j*_ ∈ *X*_*i*_
*POA*_*j*_	the *j*-th pathological object area in a *CI* and *j* ∈ [1, |*POA*|]
*NPOA*	the non-POA part of the *CI*
*CIB*_*ij*_	the *j*-th correlated image block of the POAs in *CI*_*i*_
*NIB*_*ij*_	the *j*-th image block of the NPOA part in *CI*_*i*_
*sim*(*X*_*i*_, *X*_*j*_)	similarity between two *CIS*s (i.e., *X*_*i*_ and *X*_*j*_) (ref. Eq (4))
*d*(*CI*_*i*_, *CI*_*j*_)	Euclidean distance between two *CI*s (i.e., *CI*_*i*_ and *CI*_*j*_)
*MaxN*	maximal number of the *CIS*s buffered at *N*_*E*_
*δ*	granularity value for the size of image blocking

**Fig 2:**
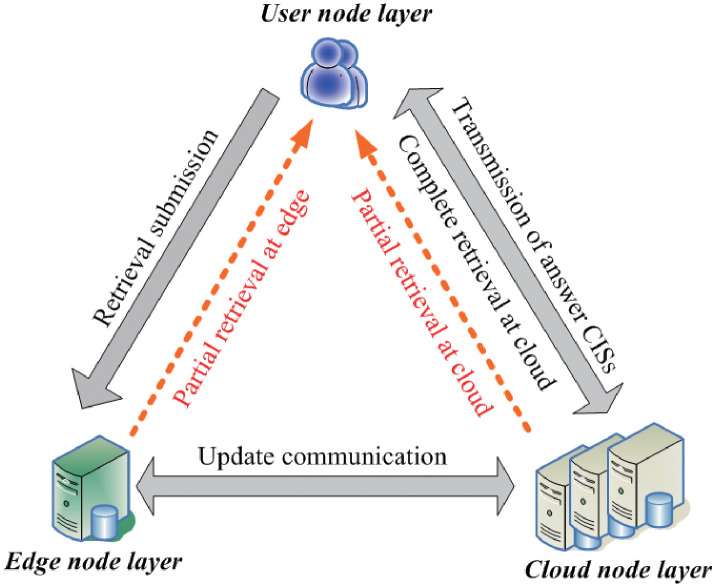
The three layer architecture of the *BRS* system.

Fig 2 depicts the three-layer network architecture of the *BRS* system, which is formally stated in Definition 1.

Definition 1(*MECN*). *A mobile edge-cloud network(MECN) is represented by a graph* (G) *which can be modeled by a triplet*:

$$\begin{array}{c} G=<N,E,T> \end{array}$$ (1)

*where*

• *N means a set of nodes, formally represented as N = N*_U_ ∪ *N*_*E*_ ∪ *N_C_, in which*

*i) N_U_ represents a user node that is used for: 1) submitting the retrieval; 2) decryption of the RIBs; 3) acquisition, reconstruction and display of the CISs*;

*ii) N_E_ represents the edge nodes that are used for: 1) temporally storing the CISs buffered at N_E_; and 2) sending back the partial answer CISs to N_U_*;

*iii) N_C_ represents the cloud nodes which are used for: 1) partition processing of the IBs; 2) encryption and storage of the CIBs; storing the NIB replicas, and 3) sending back the answer CISs to N_U_*;

• *E denotes a collection of edges representing the different network bandwidths for data communication at time T, formally denoted as: E* = < *e*_1_, *e*_2_, …, *e*_|*E*|_ >, *where e*_*k*_ = (*N*_*i*_, *N*_*j*_) *refers to the k-th edge in* G *in which N_i_ and N_j_ are connected*.

Definition 2 (*POA*). *A pathological object area (POA) in a CI can be modeled by a two-tuple*:

$$\begin{array}{c} POA_{i}=<i,PO> \end{array}$$ (2)

*where i is the ID number of the POA, PO is the coordinate of the POA in the CI*.

According to Definition 2, a non-POA part of a *CI* is denoted as *NPOA*.

Definition 3 (*IB*). *An image block (IB) can be modeled as a triplet*:

$$\begin{array}{c} IB=<bid,PO,TP> \end{array}$$ (3)

*where bid refers to the block ID, PO is the coordinate of the IB in the CI, and TP is the transmission priority of the block*.

Definition 4 (*CIB*). *Given a POA(i.e., POA_k_) in a CI, a correlated image block (CIB) of POA_k_ is an IB which is contained in or intersects with it, formally denoted as: CIB* = {*IB*_*bid*_|*IB*_*bid*_ ∩ *POA*_*k*_ ≠ ∅}, *where k* ∈ [1, |*POA*|] *and* |*POA*| *means the number of the POAs in the CI*.

Definition 5 (*NIB*). *A NIB is an IB that is contained by a NPOA in a CI, formally represented by: NIB* = {*IB*_*bid*_|*IB*_*bid*_ ∩ *NPOA* = *IB*_*bid*_}.

**Fig 3:**
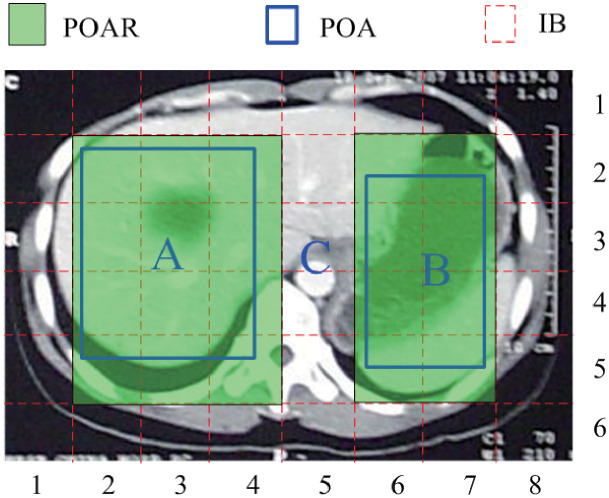
The 20 *CIB*s in a *CI* (*δ* = 6 × 8).

As indicated in introduction section, there are usually some lesion tissues that the doctors may focus on in the *CIS*s. The region of such lesion organ in the *CIS* is called the *pathological object area* (POA). In the preprocessing step, the POAs need to be preliminarily marked by medical specialists; then each *CI* in the sequence is equally divided into some *IB* (i.e., *NIB* and *CIB*) replicas, with the *CIB*s being encrypted and saved at their original pixel resolutions while the *NIB*s are stored at a lesser resolution. As illustrated in Fig 3, there are two POAs (*A* and *B*) and one NPOA (i.e., *C*) in an example *CI* which can be segmented into 6 × 8 *IB*s marked by red dash lines.

## Methodologies

In this section, we first introduce four supporting techniques based on which a *BRS* algorithm is proposed next.

### Supporting techniques

To better facilitate the batch retrieval of the lung *CIS*s in the *MECN*, in this subsection, we introduce four supporting techniques: 1) *batch similarity measure for CISs*, 2) *CIB-based privacy preserving scheme*, 3) *uniform edge-cloud index framework*, and 4) *edge buffering*.

#### Batch similarity measure for CISs

As mentioned before, a *CIS* *X*_*i*_ is a time-series data which can be modeled by a vector: *X*_*i*_ = {*CI*_1_, *CI*_2_, …, *CI*_|*X*_*i*_|_}, where |*X*_*i*_| means the number of *CI*s in *X*_*i*_. Due to the large amount of the *CI*s in a *CIS*, to effectively reduce the high computation cost in the *CIS* similarity matching, we propose a representative *CI*(*RCI*)-based batch similarity measurement of the *CIS*s.

Before introducing the batch similarity measure, how to extract the *RCI*s is a challenging issue. As summarized in Algorithm 1, given a *CIS* *X*_*i*_, a *RCI* extraction processing of *X*_*i*_ is first performed to obtain ||*X*_*i*_|| *RCI*s from a *CIS*, where ||*X*_*i*_|| means the number of *RCI*s in *X*_*i*_, *d*(*x*, *y*) is stated in Table 1, and *ε* is a small positive threshold.

**Algorithm 1** ***RCI extraction*** (*X*_*i*_)

**input**: *X*_*i*_

**output**: ||*X*_*i*_||*RCIs*

1: j←1, ||*X*_*i*_||←1;

2: **while** (*i* < |*X*_*i*_|) **do**

3:  **if** *d*(*CI*_*j*_, *CI*_*j*+1_) > *ε* **then**

4:   add *CI*_*j*+1_ as the ||*X*_*i*_||-th *RCI*;

5:   ||*X*_*i*_||++;

6:  **else**

7:   j++;

8:  **end if**

9: **end while**

10: **return** ||*X*_*i*_|| *RCI*s

Once the ||*X*_*i*_|| *RCI*s are extraction from *X*_*i*_, *X*_*i*_ can be re-represented as: $X_{i}=\{RCI_{1},RCI_{2},\ldots ,RCI_{\left||X_{i}|\right|}\}$. So given two *CIS*s (*X*_*m*_ and *X*_*n*_), their batch similarity can be defined as follows:

$$\begin{array}{c} sim\left(X_{m},X_{n}\right)=\frac{\sum_{RCI_{j}\in X_{m}}1_{\left\{RCI_{j}\in X_{n}|d\left(RCI_{i},RCI_{j}\right)\leq \epsilon \right\}}+\sum_{RCI_{i}\in X_{n}}1_{\left\{RCI_{i}\in X_{m}|d\left(RCI_{i},RCI_{j}\right)\in \epsilon \right\}}}{|X_{m}\left|+\right|X_{n}|} \end{array}$$ (4)

As can be seen from Eq (4), the similarity of two *CIS*s can be measured by the percentage of similar *RCI*s in the two corresponding *CIS*s.

#### CIB-based privacy-preserving scheme

Before introducing the *CIB*-based privacy-preserving scheme, let’s first give a definition.

Definition 6 (*POAR*). *Given a POA(i.e., POA_j_), its corresponding POA-related region (POAR) consists of all CIBs in POA_j_, subjecting to the following criteria*:

$$\begin{array}{c} \left\{ \begin{array}{c} POAR\left(POA_{j}\right)\cap POA_{j}=POA_{j}\\ |POAR\left(POA_{j}\right)|=|POA_{j}| \end{array}\right. \end{array}$$ (5)

*where POAR(POA_j_) means the corresponding POAR of POA*_*j*_, |•| *denotes the number of CIBs in* •.

In Fig 3, there are two POAs (i.e., *A* and *B*) in the *CI* that is equally segmented into 6 × 8 *IB*s. Based on Definition 6, the corresponding POARs of the two POAs are represented by the green shadow areas which consist of 20 *CIB*s. Since the nearby *CIB* numbers have the characteristics of continuous distribution, it is easier to use these *CIB*s to reconstruct the original *CI*. As a result, the objective of the encryption strategy is to disrupt the continuity of the ID numbers of the nearby *CIB*s in the *CI* by encoding the ID numbers of the *CIB*s such that the *CI* reconstruction is hard to perform.

So for each *CIB* in a *CI*, we first introduce a encoding scheme (*IBID*) of the ID numbers of the above *CIB*s, which is represented in Eq (6):

$$\begin{array}{c} IBID=SID\cdot c_{1}+IID\cdot c_{2}+rID\cdot c_{3}+cID \end{array}$$ (6)

where *SID* mean the ID of the *CIS* that the *CIB* belongs to, *IID* refers to the ID of the *CI* in which the *CIB* is contained, *rID* is row ID, *cID* is column ID, *c*1, *c*2, *c*3 are stretch constants and *c*1 >> *c*2 >> *c*3.

Based on the ID numbers of the *CIB*s in Eq (6), their encryption and decryption strategies are described as follows:

1) ***Encryption strategy***:

Algorithm 2 details the steps of the *CIB*-based encryption processing in which the ID numbers of the *CIB*s are encrypted, where *δ* and *ω* are two key values and *δ* < *SID*, *δ* < *IID* and *ω* < *rID*.

**Algorithm 2 *Encryption()***

**input**: *SID*, *IID*, *rID*, *cID* of a *CIB*

**output**: *IBID*: the encrypted ID number of the *CIB*

1: **if** *rID* is an odd number and *cID* is an odd number **then**

2:  *IBID* = (*SID* + *δ*) ⋅ *c*_1_ + (*IID* − *δ*) ⋅ *c*_2_ + (*rID* + *ω*) ⋅ *c*_3_ + *cID*

3: **else if** *rID* is an odd number and *cID* is an even number **then**

4:  *IBID* = (*SID* + *δ*) ⋅ *c*_1_ + (*IID* − *δ*) ⋅ *c*_2_ + (*rID* − *ω*) ⋅ *c*_3_ + *cID*

5: **else if** *rID* is an even number and *cID* is an odd number **then**

6:  *IBID* = (*SID* − *δ*) ⋅ *c*_1_ + (*IID* + *δ*) ⋅ *c*_2_ + (*rID* + *ω*) ⋅ *c*_3_ + *cID*

7: **else**

8:  *IBID* = (*SID* − *δ*) ⋅ *c*_1_ + (*IID* + *δ*) ⋅ *c*_2_ + (*rID* − *ω*) ⋅ *c*_3_ + *cID*

9 **end if**

10 **return** the encrypted *IBID*

2) ***Decryption strategy***:

Similarly, for the encrypted *CIB*s, their corresponding decryption processing is discussed in Algorithm 3.

**Algorithm 3 *Decryption()***

**input**: *SID*, *IID*, *rID*, *cID* of a *CIB*

**output**: *IBID*: the encrypted ID number of the *CIB*

1: **if** *rID* is an odd number and *cID* is an odd number **then**

2:  *IBID* = (*SID* − *δ*) ⋅ *c*_1_ + (*IID* + *δ*) ⋅ *c*_2_ + (*rID* − *ω*) ⋅ *c*_3_ + *cID*

3: **else if** *rID* is an odd number and *cID* is an even number **then**

4:  *IBID* = (*SID* − *δ*) ⋅ *c*_1_ + (*IID* + *δ*) ⋅ *c*_2_ + (*rID* + *ω*) ⋅ *c*_3_ + *cID*

5: **else if** *rID* is an even number and *cID* is an odd number **then**

6:  *IBID* = (*SID* + *δ*) ⋅ *c*_1_ + (*IID* − *δ*) ⋅ *c*_2_ + (*rID* − *ω*) ⋅ *c*_3_ + *cID*

7 **else**

8:  *IBID* = (*SID* + *δ*) ⋅ *c*_1_ + (*IID* − *δ*) ⋅ *c*_2_ + (*rID* + *ω*) ⋅ *c*_3_ + *cID*

9: **end if**

10: **return** the encrypted *IBID*

**Fig 4:**
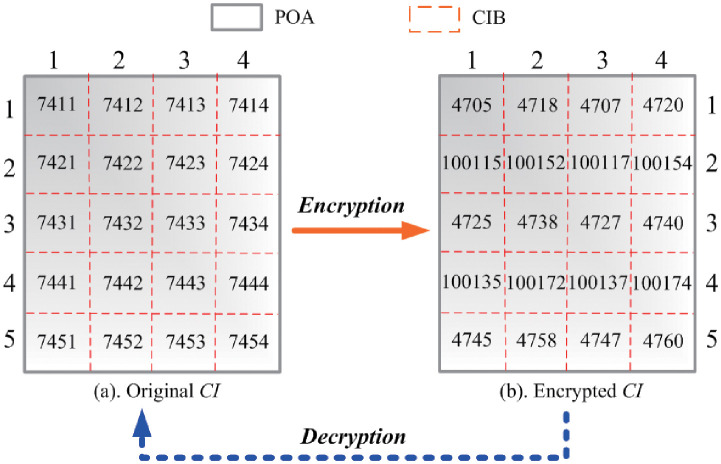
Comparison of the ID numbers of the *CIB*s before and after encryption.

For instance, assume that *SID* is 7, *IID* is 4, *c*_1_, *c*_2_, *c*_3_ are 1000, 100 and 10, respectively, then the original ID numbers of the *CIB*s before encryption are depicted in Fig 4(a). Fig 4(b) shows the encrypted ID numbers of the *CIB*s after encryption when *δ* = 3 and *ω* = 0.6.

Fig 4(a) shows the continuous distribution of the ID numbers of the nearby original *CIB*s before encryption. After encryption, as illustrated in Fig 4(b), the ID number distribution of the nearby *CIB*s is discrete. Therefore, the encryption of the *CIB*s makes it more and more difficult to find the corresponding nearby *CIB*s in the *CI* reconstruction.

Next, we proceed to analyze the probability of the successful decryption (i.e., the probability of accurate image reconstruction). Given a *CI* with *m* POAs, for each POA(i.e., *POA*_*i*_), the rows and columns of its corresponding *POAR* can be denoted as *RS*_*i*_ and *CS*_*i*_, respectively. Then, the probability that the decryption processing is successful can be derived in Eq (7):

$$\begin{array}{c} Pr\left(bDescryptSuccess=TRUE\right)=\frac{1}{\prod_{i-1}^{m}\prod_{j=0}^{RS_{i}\cdot CS_{i}-1}\left(RS_{i}\cdot CS_{i}-j\right)} \end{array}$$ (7)

Based on Eq (7), with increasing number of the *CIB*s in a *CI*, the probability of the successful decryption becomes smaller and smaller which guarantees the hardness of the decryption from a theoretical level. The encrypted *CIB*s are stored in *N*_*C*_ or *N*_*E*_ which ensures the corresponding *CIB*s’ IDs in a *CI* presents a discrete distribution rather than continuity to a certain extent. The reconstruction and display of the *CI*s are conducted at *N*_*U*_ by reversely decrypting the ID numbers of the *CIB*s based on the key values (i.e., *δ* and *ω*).

#### Uniform edge-cloud indexing framework

To support faster *CIS* filtering processing, we propose a *uniform edge-cloud index framework* (UECIF) based on iDistance [35], in which the UECIF is composed of two types of indexes: the *eIndex* in *N*_*E*_ and the *cIndex* in *N*_*C*_.

• ***Index Construction***

For the *eIndex*, initially, suppose that the *CIS*s in Ω are virtually stored in *N*_*E*_, which means the *CIS*s in Ω are physically stored in *N*_*E*_, they are logically, however, not buffered in *N*_*E*_. Then, the *CIS*s are first grouped into the *K* clusters by the AP-cluster [36] based on visual similarity (i.e., Eq (4)). Given a *CIS* *X*_*i*_, its index key can be represented below:

$$\begin{array}{c} key\left(X_{i}\right)=j\cdot c_{1}+sim\left(X_{i},X_{r}^{j}\right) \end{array}$$ (8)

where $X_{r}^{j}$ is the cluster centre of the *j*-th cluster that *X*_*i*_ belongs to, *sim*(⋅, ⋅) represents the visual similarity distance function (i.e., Eq (4)), *j* ∈ [1, *K*], and the constant *c*_1_ is used to stretch the value range.

The index key is inserted into an improved *B*^+^-Tree in which a leaf node (*LNode*) can be modeled by a triplet: *LNode*(*X*_*i*_) = < *key*, *value*, *EType* >, where *EType* = ‘F’ means *X*_*i*_ is not buffered in *N*_*E*_; otherwise, *X*_*i*_ has been buffered in *N*_*E*_. Algorithm 4 summarizes the initial construction process of the *eIndex* in which *LNode*(*X*_*i*_).*EType* ← ‘F’(line 6) means all of the *CIS*s are virtually stored in *N*_*E*_.

**Algorithm 4 *eIndex construction***(Ω)

**input**: Ω: the *CIS* set

**output**: *eIdx*

1: *eIdx*←NULL;      ▹ initialize

2: the *CIS*s in Ω are grouped into *K* clusters;    ▹ at edge node

3: **for** each *CIS*(*X*_*i*_) in Ω **do**

4:  $key\left(X_{i}\right)=j\cdot c_{1}+sim\left(X_{i},X_{r}^{j}\right)$;

5:  insert *key*(*X*_*i*_) into an improved *B*^+^-tree(i.e., *eIdx*);

6:  *LNode*(*X*_*i*_).*EType* ← ‘F’;

7: **end for**

8 **return** the *eIdx*;

Similar to the above, for the *cIndex*, first of all, the clustering processing of the *CIS*s(Ω) in *N*_*C*_ is performed to obtain T clusters based on the above visual similarity. For a *CIS* *X*_*i*_, its index key can be derived as:

$$\begin{array}{c} KEY\left(X_{i}\right)=j\cdot c_{1}+sim\left(X_{i},X_{r}^{j}\right) \end{array}$$ (9)

where *j* ∈ [1, *T*], and other parameters and symbols are the same to that of in Eq (8). In Algorithm 5, the index key is inserted into an improved *B*^+^-Tree in which a leaf node(LNode) can be represented by a triplet: *LNode*(*X*_*i*_) = < *key*, *value*, *CType* >, where *LNode*(*X*_*i*_).*CType* ← ‘T’ means *X*_*i*_ is stored in *N*_*C*_.

**Algorithm 5 *cIndex construction***(Ω)

**input**: Ω: *the CIS set*

**output**: *cIdx*

1: *cIdx*←NULL;      ▹ initialize

2: the *CIS*s in Ω are grouped into *T* clusters;    ▹ at cloud node

3: **for** each *CIS*(*X*_*i*_) in Ω **do**

4:  $KEY\left(X_{i}\right)=j\cdot c_{1}+sim\left(X_{i},X_{r}^{j}\right)$;

5:  insert *KEY*(*X*_*i*_) into an improved *B*^+^-tree(i.e., *cIdx*);

6:  *LNode*(*X*_*i*_).*CType* ← ‘T’;

7: **end for**

8: **return** the *cIdx*;

• ***Index-Support Retrieval Processing***

**Fig 5:**
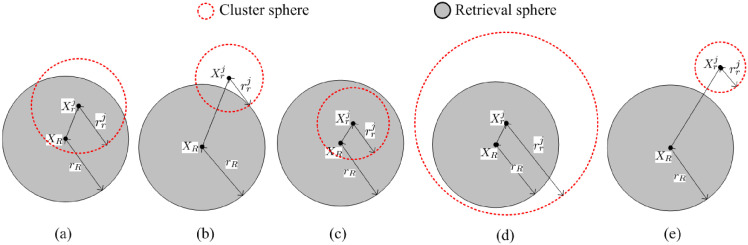
Five cases in terms of the positions of the two spheres.

Based on Eqs (8) and (9), suppose that there are *n* *CIS*s in Ω, the index keys are inserted by an improved *B*^+^-Tree respectively. So for a range retrieval Θ(*X*_*R*_, *r*_*R*_) and each cluster *C*_*j*_, as illustrated in Fig 5, there are five cases in terms of the positions of the two spheres.

Case 1: in Fig 5(a), the inequalities $sim\left(X_{r}^{j},X_{R}\right)-r_{R}<r_{r}^{j}$ and $sim\left(X_{r}^{j},X_{R}\right)<r_{R}$ are met, which means Θ(*X*_*R*_, *r*_*R*_) intersects with $\Theta (X_{r}^{j},r_{r}^{j})$ by which *X*_*R*_ is contained. So the search range is represented as: $\left[j\cdot c_{1},j\cdot c_{1}+d\left(X_{r}^{j},X_{R}\right)+r_{r}^{j}\right]$;

Case 2: in Fig 5(b), the inequalities $sim\left(X_{r}^{j},X_{R}\right)-r_{R}\leq r_{r}^{j}$ and $sim\left(X_{r}^{j},X_{R}\right)\geq r_{R}$ are met, which means Θ(*X*_*R*_, *r*_*R*_) intersects with $\Theta (X_{r}^{j},r_{r}^{j})$ and $\Theta (X_{r}^{j},r_{r}^{j})$ does not contain *X*_*R*_. So the search range is represented as: $\left[j\cdot c_{1}+sim\left(X_{r}^{j},X_{R}\right)-r_{R},j\cdot c_{1}+r_{r}^{j}\right]$;

Case 3: in Fig 5(c), the inequality $sim\left(X_{r}^{j},X_{R}\right)+r_{r}^{j}\leq r_{R}$ is met, which means Θ(*X*_*R*_, *r*_*R*_) contains $\Theta (X_{r}^{j},r_{r}^{j})$. So the search range is represented as: $\left[j\cdot c_{1},j\cdot c_{1}+r_{r}^{j}\right]$;

Case 4: in Fig 5(d), the inequality $sim\left(X_{r}^{j},X_{R}\right)+r_{R}\leq r_{r}^{j}$ is met, which means $\Theta (X_{r}^{j},r_{r}^{j})$ contains Θ(*X*_*R*_, *r*_*R*_). So the search range is represented as: $\left[j\cdot c_{1},j\cdot c_{1}+r_{r}^{j}\right]$;

Case 5: in Fig 5(e), the inequality $sim\left(X_{r}^{j},X_{R}\right)>r_{r}^{j}+r_{R}$ is met, which means Θ(*X*_*R*_, *r*_*R*_) does not intersect with $\Theta (X_{r}^{j},r_{r}^{j})$. No candidate sequences are retrieved.

For the similarity retrieval in the *MECN*, there are two cases in terms of whether there exists a partial answer in *N_E_: 1) the complete answer sequences*(Ψ) *are directly obtained from N_C_ based on cIndex (see Algorithm 8); 2) the complete answer sequences* (Ψ) *are composed of the partial answer ones* (Ψ′) *obtained from N_E_ (see Algorithm 6) and the partial answer ones*(Ψ″) *from N_C_ (see Algorithm 7)*.

Algorithm 6 details the similarity range retrieval of the *CIS*s based on the *eIndex* in *N*_*E*_. The routing ***Search()*** is the implementation of the range similarity search in the improved *B*^+^-Tree which is described in Algorithm 9.

**Algorithm 6** ***ESearch***(*X*_*R*_, *r*_*R*_, Ω′)

**input**: Θ(*X*_*R*_, *r*_*R*_): the retrieval *CIS*,

   Ω′: the *CIS* whose ETypes are ‘T’ in *N*_*E*_

**output**: Ψ′: the partial answer *CIS*s from *N*_*E*_

1: Ψ′ ← Φ;      ▹ initialization

2: Ψ′ ← ***Search***(*X*_*R*_, *r*_*R*_, Ω′)

3: **for** each candidate *CIS*(*X*_*i*_) ∈ Ψ′ **do**

4:  **if** *sim*(*X*_*R*_, *X*_*i*_) > *r*_*R*_ **then**

5:   Ψ′ ← Ψ′ − *X*_*i*_;

6:  **else**

7:   **if** *LNode*(*X*_*i*_).*EType* = ‘F’ **then**

8:    *LNode*(*X*_*i*_).*EType* ← ‘T’;      ▹ for *eIndex*

9:    *LNode*(*X*_*i*_).*CType* ← ‘F’;      ▹ for *cIndex*

10:    update the information of *X*_*i*_ (e.g., access frequencies and access time) in the log file;

11:   **end if**

12:  **end if**

13: **end for**

14: **return** Ψ′

Similarly, Algorithm 7 summarizes the index support partial similarity range retrieval of the *CIS*s at the cloud node level. It is worth mentioning that *LNode*(*X*_*i*_).*CType* = ‘T’ means *X*_*i*_ is not buffered in *N*_*E*_.

**Algorithm 7 *CSearch***(*X*_*R*_, *r*_*R*_, Ω′)

**input**: Θ(*X*_*R*_, *r*_*R*_): the retrieval *CIS*,

   Ω′: the *CIS* whose ETypes are ‘T’ in *N*_*c*_

**output**: Ψ′: the partial answer *CIS*s from cloud node

1: Ψ′ ← Φ;      ▹ initialization

2: Ψ′ ← ***Search***(*X*_*R*_, *r*_*R*_, Ω′)

3: **for** each candidate *CIS*(*X*_*i*_) ∈ Ψ′ **do**

4:  **if** *sim*(*X*_*R*_, *X*_*i*_) > *r*_*R*_ **then**

5:   Ψ′ ← Ψ′ − *X*_*i*_;

6:  **else**

7:   **if** *LNode*(*X*_*i*_).*EType* = ‘F’ **then**

8:    *LNode*(*X*_*i*_).*EType* ← ‘T’;      ▹ for *eIndex*

9:    *LNode*(*X*_*i*_).*CType* ← ‘F’;      ▹ for *cIndex*

10:   **end if**

11:  **end if**

12: **end for**

13: **return** Ψ′

Finally, obtaining the complete answer *CIS*s from the cloud node is detailed in Algorithm 8.

**Algorithm 8 *DSearch***(*X*_*R*_, *r*_*R*_, Ω)

**input**: Θ(*X*_*R*_, *r*_*R*_): the retrieval *CIS*,

   Ω: the *CIS* in *N*_*c*_

**output**: Ψ: the complete answer *CIS*s from *N*_*c*_

1: Ψ ← Φ      ▹ initialization

2: Ψ ← ***Search***(*X*_*R*_, *r*_*R*_, Ω)

3 **for** each (*X*_*i*_) ∈ Ψ **do**

4:  **if** *sim*(*X*_*R*_, *X*_*i*_) > *r*_*R*_ **then**

5:   Ψ ← Ψ − *X*_*i*_;

6:  **end if**

7: **end for**

8: **return** Ψ

**Algorithm 9 *Search***(*X*_*R*_, *r*_*R*_, Ω)

**input**: Θ(*X*_*R*_, *r*_*R*_): the retrieval *CIS*,

   Φ: the *CIS*s

**output**: Φ′: the candidate *CIS*s

1: Φ′ ← *NULL*;

2: **for** the *CIS*s in each cluster *C*_*j*_ **do**

3:  **if** $sim\left(X_{R},X_{i}\right)-r_{R}<r_{r}^{j}$ and $sim\left(X_{r}^{j},X_{R}\right)<r_{R}$ **then**

4:   $left=j\cdot c_{1},right=j\cdot c_{1}+sim\left(X_{r}^{j},X_{R}\right)+r_{r}^{j}$;

5:  **else if** $sim\left(X_{R},X_{i}\right)-r_{R}\leq r_{r}^{j}$ and $sim\left(X_{r}^{j},X_{R}\right)\geq r_{R}$ **then**

6:   $left=j\cdot c_{1}+sim\left(X_{r}^{j},X_{R}\right)-r_{R},right=j\cdot c_{1}+r_{r}^{j}$;

7:  **else if** $sim\left(X_{r}^{j},X_{R}\right)+r_{r}^{j}\leq r_{R}$ **them**

8:   $left=j\cdot c_{1},right=j\cdot c_{1}+r_{r}^{j}$;

9:  **else if** $sim\left(X_{r}^{j},X_{R}\right)+r_{R}\geq r_{r}^{j}$ **then**

10:   $left=j\cdot c_{1},right=j\cdot c_{1}+r_{r}^{j}$;

11:  **else**

12:   exit();

13:  **end if**

14:  Φ′ ← Φ′ ∪ ***BRSearch***[*left*, *right*];

15: **end for**

16: **return** Φ′

• ***Index Update***

When user submits a retrieval request, the *eIndex* needs to be updated by adding the *CIS*s that have been accessed in this retrieval. Since the number of *CIS*s buffered in *N*_*E*_ is limited, how to optimally choose the buffered *CIS*s is challenging.

For example, assume that there are six *CIS*s in *N*_*E*_, Tables 2 and 3 illustrate the ranking of *access time* (AT) and *access frequencies* (AF) for the six *CIS*s, respectively. In Table 2, the ATs of the six *CIS*s are sorted in an ascending order, which are quantitatively represented by the *AT_ID*s. Then a *weighted* AT (WAT) can be derived as follows:

$$\begin{array}{c} WAT\left(CIS_{j}\right)=\frac{AD\_ID_{j}}{{\text{argMax}}_{i\in [1,n]}\left(AT\_{ID}_{i}\right)} \end{array}$$ (10)

**Table 2:** Ranking of the AT.

	*CIS*_1_	*CIS*_2_	*CIS*_3_	*CIS*_4_	*CIS*_5_	*CIS*_6_
*AT*	2:50	3:40	1:48	4:21	2:32	3:11
*AT_ID*	4	2	6	1	5	3
*WAT*	4/6	2/6	6/6	1/6	5/6	3/6

**Table 3:** Ranking of the AF.

	*CIS*_1_	*CIS*_2_	*CIS*_3_	*CIS*_4_	*CIS*_5_	*CIS*_6_
*AF*	1	3	1	4	2	3
*WAF*	4/5	2/5	4/5	1/5	3/5	2/5

Similarly, for the ranking of the *access frequencies*(AF), a *weighted* AF(WAF) is represented in Eq (11):

$$\begin{array}{c} WAF\left(CIS_{j}\right)=1-\frac{AF_{j}}{{\text{argMax}}_{i\in [1,n]}\left({AF}_{i}\right)} \end{array}$$ (11)

Based on Eqs (10) and (11), given a *CIS*_*j*_, its uniform ranking score (*URS*) is shown below:

$$\begin{array}{c} URS\left(CIS_{j}\right)=WRA\left(CIS_{j}\right)+WAF\left(CIS_{j}\right) \end{array}$$ (12)

Table 4 illustrates the uniform ranking scores of the six *CIS*s. Based on Eq (12), the smaller the *URS*, the more important the *CIS*. If *MaxN* is 4, then *CIS*_3_ and *CIS*_1_ can be removed from the edge buffer.

**Table 4:** Uniform ranking score.

	*CIS*_1_	*CIS*_2_	*CIS*_3_	*CIS*_4_	*CIS*_5_	*CIS*_6_
*WRA*	4/6	2/6	6/6	1/6	5/6	3/6
*WAF*	4/5	2/5	4/5	1/5	3/5	2/5
*URS*	44/30	22/30	54/30	11/30	43/30	27/30

**Algorithm 10 *Update***(Ω′)

**input**: Ω′: the *CIS*s buffered at *N*_*E*_

**output**: the updated Ω′

1: **for** *i* = 1 to *MaxN* − |Ω′| − 1 **do**

2:  remove a *CIS*_*j*_ whose URS is the largest from Ω′;

3:  Ω′ ← Ω′ − *CIS*_*j*_;

4:  *LNode*(*CIS*_*j*_).*CType* ← ‘T’;

5:  *LNode*(*CIS*_*i*_).*EType* ← ‘F’;

6: **end for**

7: **return** the updated Ω′

### Edge buffering

Unlike the traditional image retrieval methods, which directly obtain data from the remote cloud, if the answer *CIS*s can be directly obtained from the edge without accessing the cloud, it will greatly shorten the long-distance transmission delay and improve the retrieval efficiency. Based on the above motivation, we propose an edge buffering scheme by analyzing the user historical retrieval (HR) log file. The refinement cost of the candidate *CIS*s can be significantly decreased with the help of the buffering scheme since a portion of answer *CIS*s can be retrieved directly without any refinement processing.

Specifically, assume that *n* HRs have been successfully completed with accurate results. Due to the fact that the answer *CIS*s provided by each HR have been verified, when a user submits a new retrieval *CIS* (i.e., *X*_*R*_), it is highly possible that *X*_*R*_ may be similar or even the same as the HR one (i.e., $X_{R}^{\prime i}$). As a result, the retrieval efficiency and accuracy can be greatly improved if the HR results in *N*_*E*_ can be carefully reused as a part of the current results.

Definition 7(*CRS*). *Given a retrieval* CIS *X_R_ and a retrieval radius r_R_, their corresponding* CIS *retrieval sphere (*CRS*) is a high-dimensional sphere with a centre X_R_ and a radius r_R_, denoted as* Θ(*X*_*R*_, *r*_*R*_).

Definition 8(*HCRS*). *Given a HR* CIS $X_{R}^{\prime i}$ *and a retrieval radius* $r_{R}^{\prime i}$, *their corresponding historical* CIS *retrieval sphere (HCRS) is a high-dimensional sphere with a centre* $X_{R}^{\prime i}$ *and a radius* $r_{R}^{\prime i}$, *denoted as* $\Theta (X_{R}^{\prime i},r_{R}^{\prime i})$.

Definition 9(*AA*). *Given a* CRS Θ(*X*_*R*_, *r*_*R*_) *and a HCRS* $\Theta (X_{R}^{\prime i},r_{R}^{\prime i})$, *their corresponding affected area (AA) is the intersection part of the two spheres, formally denoted as*: $AA(\Theta \left(X_{R},r_{R}\right)\cup \Theta \left(X_{R}^{\prime i},r_{R}^{\prime i}\right))$.

**Fig 6:**
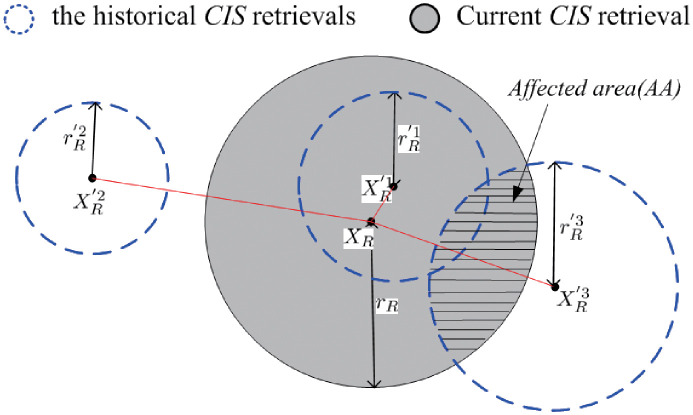
An example of the edge buffering scheme.

For example, as shown in Fig 6, there are three *HCRS*s, i.e., $\Theta (X^{\prime 1},r_{R}^{\prime 1})$, $\Theta (X_{R}^{\prime 2},r_{R}^{\prime 2})$ and $\Theta (X_{R}^{\prime 3},r_{R}^{\prime 3})$. The current *CRS* is represented as: Θ(*X*_*R*_, *r*_*R*_). For *X*_*R*_, it’s corresponding 1_*st*_, 2_*nd*_ and 3_*rd*_ nearest neighbor *CIS*s are $X_{R}^{\prime 1}$, $X_{R}^{\prime 3}$ and $X_{R}^{\prime 2}$, respectively. Therefore, the *HR* of $\Theta (X^{\prime 2},r_{R}^{\prime 2})$ can be safely discarded since its corresponding *HCRS* does not intersect with Θ(*X*_*R*_, *r*_*R*_). The *CIS*s falling in the AA (i.e., $\Theta \left(X_{R},r_{R}\right)\cup \Theta \left(X_{R}^{\prime 1},r_{R}^{\prime 1}\right)$ and $\Theta \left(X_{R},r_{R}\right)\cup \Theta \left(X_{R}^{\prime 3},r_{R}^{\prime 3}\right)$ can be a part of the answer *CIS*s of Θ(*X*_*R*_, *r*_*R*_).

**Fig 7:**
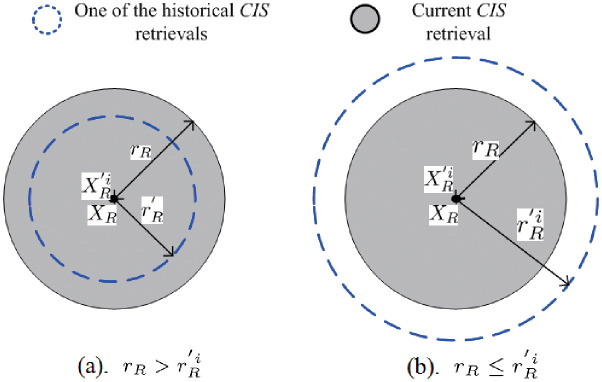
$X_{R}=X_{R}^{\prime i}$ .

**Fig 8:**
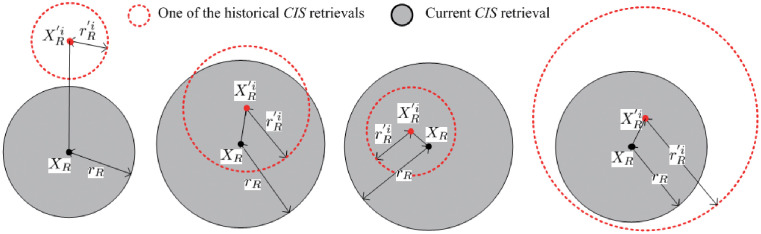
$X_{R}\neq X_{R}^{\prime i}$ , (**a**). $AA(\Theta \left(X_{R},r_{R}\right),\Theta \left(X_{R}^{\prime i},r_{R}^{\prime i}\right))=NULL$, (**b**). $AA(\Theta \left(X_{R},r_{R}\right),\Theta \left(X_{R}^{\prime i},r_{R}^{\prime i}\right))\neq NULL$, (**c**). $AA\left(\Theta \left(X_{R},r_{R}\right),\Theta \left(X_{R}^{\prime i},r_{R}^{\prime i}\right)\right)=\Theta \left(X_{R}^{\prime i},r_{R}^{\prime i}\right)$, (**d**). $AA\left(\Theta \left(X_{R},r_{R}\right),\Theta \left(X_{R}^{\prime i},r_{R}^{\prime i}\right)\right)=\Theta \left(X_{R},r_{R}\right)$.

Next, given two *CIS* retrieval spheres: Θ(*X*_*R*_, *r*_*R*_) and $\Theta (X_{R}^{\prime i},r_{R}^{\prime i})$, there exists two cases on the basis of the two retrieval *CIS*s (i.e., *X*_*R*_ and $X_{R}^{\prime i}$), which are shown in Figs 7 and 8, respectively.

In Fig 7, if $X_{R}=X_{R}^{\prime i}$, then there exists two cases in terms of the retrieval radii (i.e., *r*_*R*_ and $r_{R}^{\prime i}$).

• For case (a) which is formally represented as: $AA\left(\Theta \left(X_{R},r_{R}\right),\Theta \left(X_{R}^{\prime i},r_{R}^{\prime i}\right)\right)=\Theta \left(X_{R}^{\prime i},r_{R}^{\prime i}\right)$, since the *CIS*s falling in the *HCRS* $\Theta (X_{R}^{\prime i},r_{R}^{\prime i})$ have already undergone verification, they can be part of the answer *CIS*s for Θ(*X*_*R*_, *r*_*R*_);

• For case (b) which is formally represented as: $AA\left(\Theta \left(X_{R},r_{R}\right),\Theta \left(X_{R}^{\prime i},r_{R}^{\prime i}\right)\right)=\Theta \left(X_{R},r_{R}\right)$, the answer *CIS*s for Θ(*X*_*R*_, *r*_*R*_) can be derived from the *CIS*s in $\Theta (X_{R}^{\prime i},r_{R}^{\prime i})$.

In Fig 8, if $X_{R}\neq X_{R}^{\prime i}$, there are four cases according to the placement of the two spheres (i.e., Θ(*X*_*R*_, *r*_*R*_) and $\Theta (X_{R}^{\prime i},r_{R}^{\prime i})$).

• In case (a), as the *AA* of the above two spheres does not exist, formally represented as: $AA(\Theta \left(X_{R},r_{R}\right),\Theta \left(X_{R}^{\prime i},r_{R}^{\prime i}\right))=NULL$, the answer *CIS*s need to be calculated sequentially in Θ(*X*_*R*_, *r*_*R*_);

• In case (b), as the *AA* of the above two spheres exists, formally represented as: $AA(\Theta \left(X_{R},r_{R}\right),\Theta \left(X_{R}^{\prime i},r_{R}^{\prime i}\right))\neq NULL$. Since the *CIS*s falling in $\Theta (X_{R}^{\prime i},r_{R}^{\prime i})$ have been verified previously, they can be regarded as a part of a candidate *CIS* set of Θ(*X*_*R*_, *r*_*R*_);

• In case (c), as the *AA* of the above two spheres is $\Theta (X_{R}^{\prime i},r_{R}^{\prime i})$, formally represented as: $AA\left(\Theta \left(X_{R},r_{R}\right),\Theta \left(X_{R}^{\prime i},r_{R}^{\prime i}\right)\right)=\Theta \left(X_{R}^{\prime i},r_{R}^{\prime i}\right)$. As the *CIS*s that fall in $\Theta (X_{R}^{\prime i},r_{R}^{\prime i})$ have been verified previously; they can be regarded as a part of an answer *CIS* set of Θ(*X*_*R*_, *r*_*R*_);

• In case (d), as the *AA* of the above two spheres is Θ(*X*_*R*_, *r*_*R*_), formally represented as: $AA\left(\Theta \left(X_{R},r_{R}\right),\Theta \left(X_{R}^{\prime i},r_{R}^{\prime i}\right)\right)=\Theta \left(X_{R},r_{R}\right)$, the answer *CIS*s are contained by the *CIS*s falling in $\Theta (X_{R}^{\prime i},r_{R}^{\prime i})$.

### The BRS algorithm

**Fig 9:**
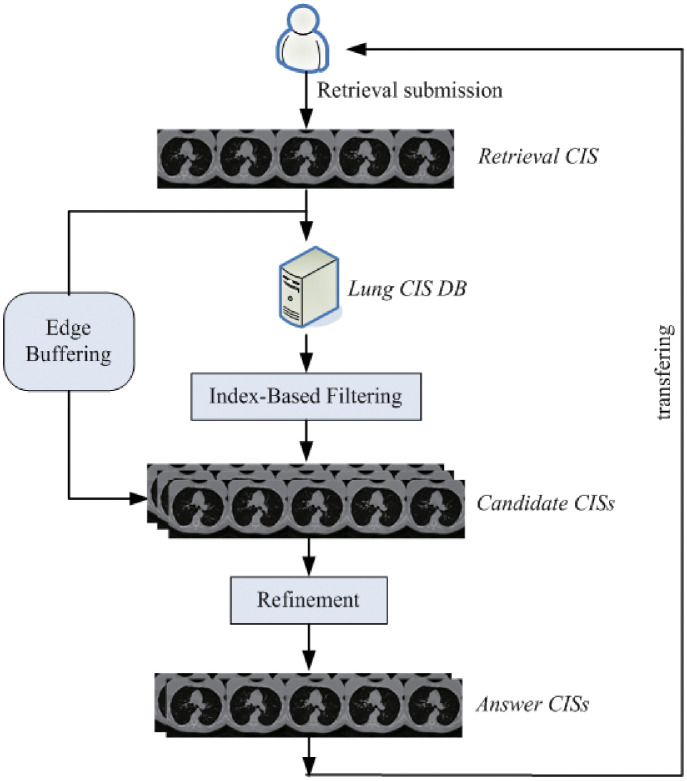
An example of the *BRS* processing.

Before introducing the algorithm, a pre-processing step is first conducted. Algorithm 11 summarizes the detailed steps of our proposed *BRS* method in which ***ESearch***(*X*_*R*_,*r*_*R*_), ***CSearch***(*X*_*R*_,*r*_*R*_) and ***DSearch***(*X*_*R*_,*r*_*R*_) correspond to Algorithms 6-8, respectively. As illustrated in Fig 9, first of all, when a retrieval lung *CIS* (*X*_*R*_) is submitted to the edge node level *N*_*E*_ from the user one *N*_*U*_, then the *eIndex* scheme in *N*_*E*_ is adopted to quickly judge whether there are some answer *CIS*s similar to *X*_*R*_. If exists, then the high-dimensional similarity retrieval is carried out with the support of the *cIndex* scheme at the cloud to obtain some partial retrieval answer *CIS*s; otherwise, the similarity retrieval of all *CIS*s supported by the *cIndex* is performed directly through the cloud, and finally the answer *CIS*s are returned to the receiver node. Note that, in line 9, before transmitting the answer *CIS*s to the receiver, the decryption processing of the *CIB*s in the *CI*s need to be performed to ensure the accurate reconstruction and display of the answer *CIS*s. Compared to *NIB*s, the *CIB*s have higher transmission priorities. In accordance with the various priorities of the *IB*s, they can be transmitted in descending order of priority, which not only assures the security of data transmission but also ensures that the critical information can be transmitted first.

**Algorithm 11 *BRS***(*X*_*R*_, *r*_*R*_)

**input**: *X*_*R*_: a retrieval *CIS*, *r*_*R*_: a retrieval radius

**output**: Ψ: the answer *CIS*s

1: a retrieval *CIS* (*X*_*R*_) is submitted from *N*_*U*_;

2: Ψ′ ← ***ESearch***(*X*_*R*_, *r*_*R*_); ▹ obtain answer *CIS*s(Ψ′) based on the *eIndex* at the edge node *U*_*E*_;

3: Ψ′ ≠ *NULL* **then**

4:  Ψ″ ← ***CSearch***(*X*_*R*_, *r*_*R*_); ▹ obtain the partial answer *CIS*s(Ψ″) based on *cIndex* at the cloud *U*_*C*_;

5:  Ψ ← Ψ′ ∪ Ψ″;

6: **else**

7:  Ψ ← ***DSearch***(*X*_*R*_, *r*_*R*_); ▹ obtain the complete answer *CIS*s based on *cIndex* at the cloud *U*_*C*_;

8: **end if**

9: transmit the *CIS*s in Ψ to the receiver node level with different transmission priorities

### Experiments

To verify the efficiency of the proposed *BRS* method, extensive simulation experiments are conducted to demonstrate the retrieval performance.

#### Experimental setup

In the experiments, the image receiver client is equipped with a 5.9-inch, full HD 1080p screen and a Qualcomm^®^ Snapdragon^™^ 650 processor running at 1.7GHz. The client system is developed in Java and operates on the Android operating system [37]. The edge node and the cloud one are connected via 1Gbps network links. In the cloud node, the *IB*s (i.e., *CIB* and *NIB*) with various transmission priorities are kept in a file system and some structured data is recorded by the MySQL [38]. Each node contains a 2.7 GHz quad-core Xeon processor, 2.0 Gigabyte memory, and 1.0 Terabyte hard disk. The maximum data communication rate is 150 Mbps in the wireless network.

We selected the LUNA16 dataset [39], which contains 239,232 lung *CI*s, as our experimental dataset. There are 888 lung *CIS*s in this database, with an average of 336 lung *CIS*s each set. The lung *CIS*s in each set range in level from 200 to 600.

#### A prototype retrieval system

**Fig 10:**
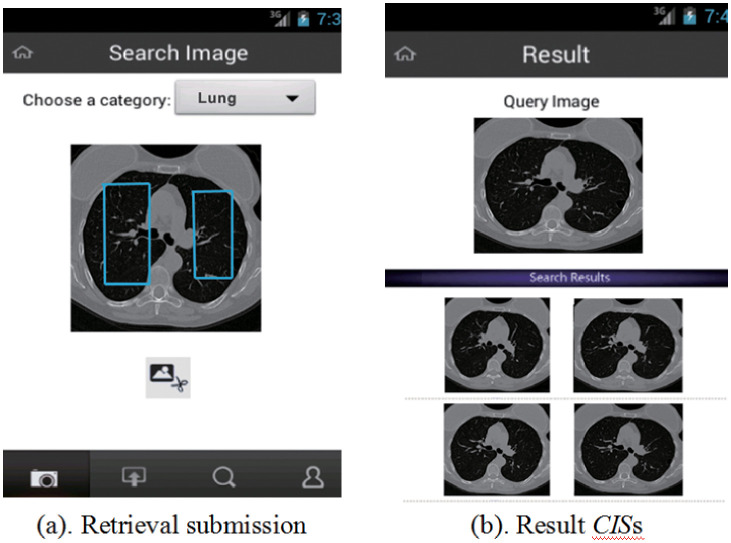
A prototype system of the *BRS*.

Fig 10 depicts a demonstration of the prototype system. An example of the *CIS* pre-processing backend interface is shown in Fig 10(a) in which a POA as been marked by a blue rectangle line. In Fig 10(b), a *CIS* with the category ‘*lung*’ has been inputted as a retrieval sequence. Four result *CIS*s were quickly retrieved, and their matching *IB*s are restored and shown.

#### Effectiveness of the *BRS* method

The first experiment testifies the effectiveness of our *BRS* method by using the lung *CIS*s randomly selected as experimental data. The recall and precision achieved by this retrieval method can be defined as:

$$\begin{array}{c} recall=\frac{\left|rel\cap ret\right|}{\left|ret\right|},\;precision=\frac{\left|rel\cap ret\right|}{\left|rel\right|} \end{array}$$ (13)

where *rel* means the set of ground-truth, and *ret* refers to the set of results returned by a similarity range search.

**Fig 11:**
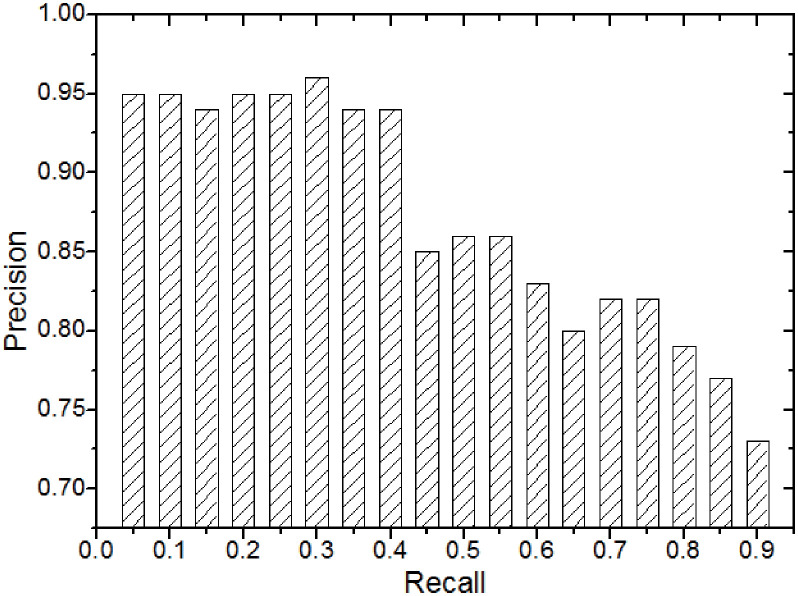
Retrieval accuracy.

As shown in Fig 11, performance comparisons of the retrieval effectiveness of the 10 *CIS*s with the same organ (i.e., *lung*) that are randomly selected from the database are conducted. As can be observed from the figure, precision steadily declines as recall ratio rises. The reason is that when the recall rate is low, it’s highly possible that the correctness rate of the result *CIS*s is high. Meanwhile, the high recall rate can not guarantee that the retrieval results contain the correct *CIS*s.

#### Effect of data size

**Fig 12:**
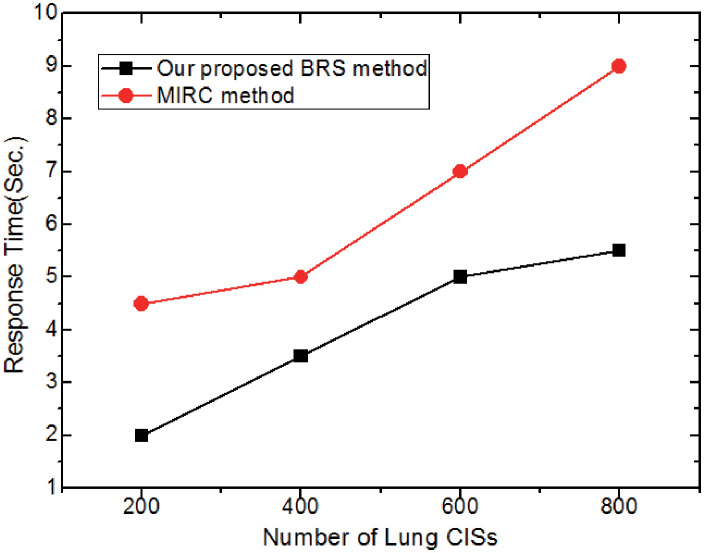
Effect of data size.

In this experiment, we investigate the effect of data size (i.e., the number of the lung *CIS*s) on the retrieval efficiency by using the two methods: 1) our proposed *BRS* method; 2) The *MIRC* method in [25]. In this experiment, the network bandwidth is 100Mbps and the number of edge nodes is 15, and the *UECI* framework is used. In Fig 12, with the increase of the *CIS*s, the *BRS* method is superior to the *MIRC* since the edge buffer is verified to significantly reduce the retrieval computation cost and transmission delay. Meanwhile, it is interesting to observe that as the data size increases, the overall response time first grows rapidly and then gradually. This is because the index performs better when there is more data.

#### Effect of *ε*

**Fig 13:**
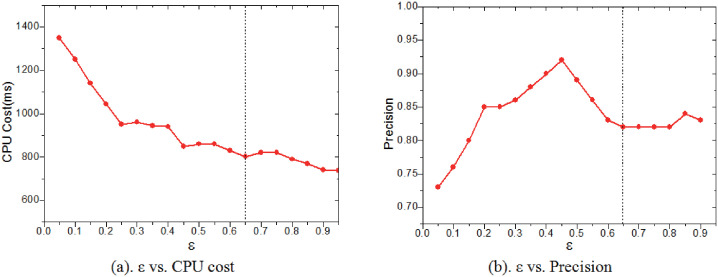
Effect of *ε*.

The experiment evaluates the effect of *ε* on the retrieval performance. Similar to the above experiment, the network bandwidth and the number of cloud(edge) nodes are fixed, and the edge buffering scheme and the indexing mechanism are adopted. As demonstrated in Fig 13(a), with the increase of *ε*, the CPU cost for the similarity computation is decreasing due to the decrease of the number of the *RCI*s in each *CIS*. Meanwhile, it’s interesting to note in Fig 13(b) that the precision ratio increases rapidly first and then decreases gradually. The reason is that too many or too few *RCI*s will make it difficult to accurately and completely measure the similarity of the *CIS*s. Therefore, an optimal *ε* is set to be 0.65.

#### Effect of edge buffering scheme

**Fig 14:**
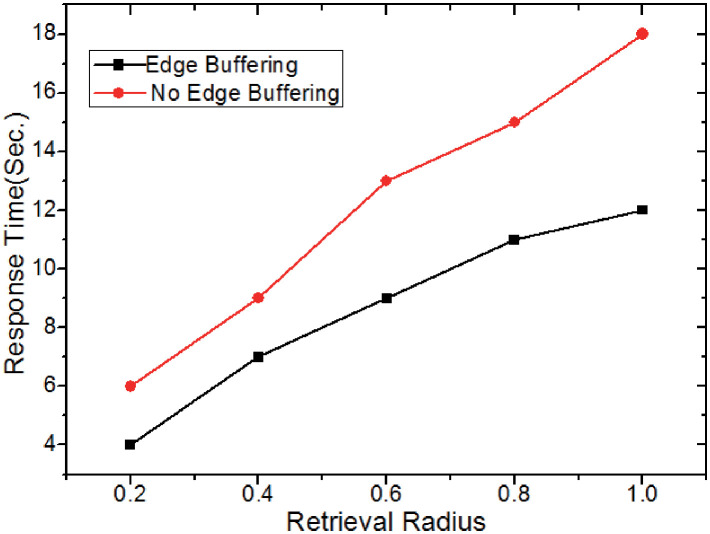
Effect of edge buffering scheme.

In this experiment, we proceed to study the effect of the edge buffering scheme on the retrieval performance. Method 1 adopts the edge buffering scheme and method 2 do not use it. Fig 14 demonstrates that the overall response time using method 1 is faster than method 2 when the bandwidth is stable and the retrieval radius (*r*_*R*_) is fixed. Meanwhile, the performance gap widens as *r*_*R*_ steadily grows while the band-width remains constant. This is because with the increase of *r*_*R*_, the probability of obtaining the result *CIS*s in the edge buffering is also increasing.

#### Effect of indexing scheme

**Fig 15:**
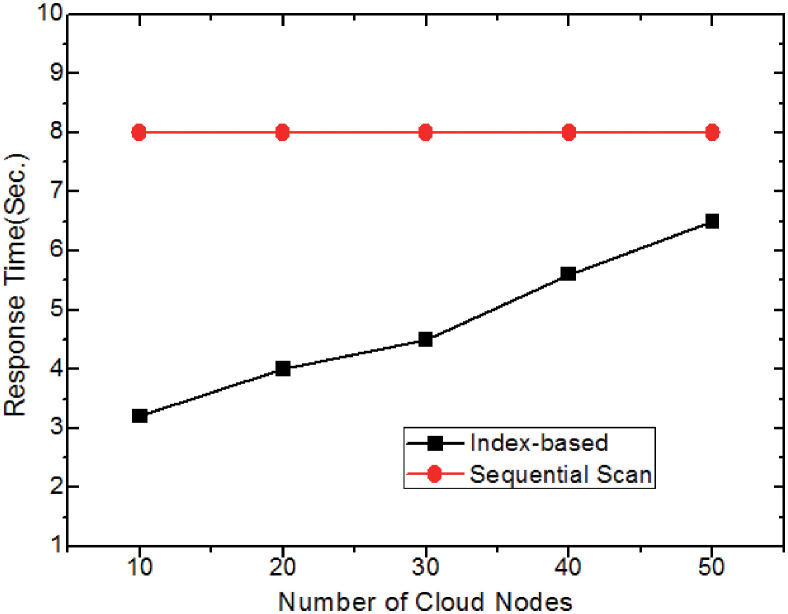
Effect of indexing scheme.

The final experiment examines how the index framework (i.e., *eIndex* and *cIndex*) affects retrieval efficiency. Here, method 1 uses the aforementioned two indexes, whereas method 2 does not (i.e., it sequentially searches each cloud node to find the answer *CIS*s). In Fig 15, when the data size and the network bandwidth are fixed, the number of the cloud nodes varies from 10 to 50, the response time for the method 1 (i.e., index-based retrieval) is growing with the number of the cloud nodes increases. Meanwhile, the performance gap of the two approaches becomes smaller since the response time for method 2 is relatively stable and locating the corresponding candidate *CIS*s based on the index is faster than that of no index. It’s interesting to notice that the retrieval response time is the smallest when the number of cloud nodes is 10. The larger the number of cloud nodes involved in retrieval, a large amount of data exchange and transmission will occur, resulting in retrieval delay.

### Conclusion

In this paper, we introduced the *BRS* method—a privacy-preserving batch retrieval of the lung *CIS*s in edge-cloud collaborative computing environment. The goal of our proposed *BRS* is to provide a safe and efficient retrieval of the lung *CIS*s in resource- constraint network with low and unstable network bandwidth. To enable the efficient *BRS* processing, four supporting techniques are proposed, namely, 1) *batch similarity measure for CISs*, 2) *CIB-based privacy preserving scheme*, 3) *uniform edge-cloud index framework*, and 4) *edge buffering scheme*. The experimental results reveal that the efficiency of the *BRS* method is more than 200% higher than that of the sequential retrieval with the aid of the supporting techniques, especially when the number of cloud nodes is smaller.
